# Perinatal and Neonatal Outcomes in Infants of Diabetic Mothers: A Prospective Descriptive Study in a Tertiary Care Center in South India

**DOI:** 10.7759/cureus.108514

**Published:** 2026-05-08

**Authors:** Jane Allen C Arokiadas, M Manjukeshwari, S Vijayalakshmi, Sridurga Mattyvanan, R Lakshmikanth

**Affiliations:** 1 Pediatrics, Vinayaka Missions Medical College and Hospital, Vinayaka Mission's Research Foundation (Deemed to be University), Karaikal, IND; 2 Community Medicine, Dhanalakshmi Srinivasan Medical College and Hospital, Siruvachur, IND; 3 Biochemistry, Vinayaka Missions Medical College and Hospital, Vinayaka Mission's Research Foundation (Deemed to be University), Karaikal, IND; 4 Medicine, Nambiyappan Hospital, Thirunelveli, IND

**Keywords:** birth weight, gestational diabetes mellitus, hyperbilirubinaemia, hypoglycaemia, neonatal outcomes, prospective study, respiratory distress

## Abstract

Background

Maternal diabetes is a well-recognized contributor to neonatal morbidity. Infants born to mothers with gestational diabetes mellitus (GDM) and pregestational diabetes are predisposed to metabolic, respiratory, and hematological disturbances. Understanding the distribution of these outcomes is important for optimizing neonatal care.

Objectives

To describe neonatal outcomes, including metabolic, respiratory, hematological, and anthropometric parameters, along with perinatal factors and maternal demographic and clinical characteristics among infants of diabetic mothers in a tertiary care setting in South India.

Methods

A prospective descriptive study was conducted over one year in a tertiary care hospital in South India. A total of 161 eligible mother-infant pairs, in which the mothers had diabetes, were included during the study period from September 2024 to August 2025. Maternal demographic, clinical, and treatment-related characteristics were recorded. Neonatal outcomes, including metabolic, respiratory, hematological, and anthropometric parameters, were assessed using standard definitions. Data were analyzed using descriptive statistics.

Results:

Among 161 infants, the majority were delivered at term (143, 88.8%). Hypoglycemia was the most frequently observed metabolic abnormality, affecting 22 (13.6%) neonates, followed by hypomagnesemia in five (3.0%) and hypocalcemia in four (2.4%). Hyperbilirubinemia was noted in 35 (21.7%) neonates. Respiratory distress was observed in 25 (15.5%) infants, with transient tachypnoea accounting for the majority. Most infants were appropriate for gestational age (124, 77.0%), while 20 (12.4%) were large and 17 (10.5%) were small for gestational age.

Conclusion

Infants of diabetic mothers demonstrated hypoglycemia, hyperbilirubinemia, and respiratory distress, which were the most common outcomes. These findings reinforce the importance of systematic surveillance and timely intervention to reduce neonatal morbidity in this high-risk population.

## Introduction

Diabetes mellitus is a metabolic disorder characterized by chronic hyperglycemia resulting from defects in insulin secretion, insulin action, or both [1]. The rising prevalence of diabetes globally has led to an increasing number of pregnancies complicated by altered glucose metabolism [2]. Gestational diabetes mellitus (GDM) and pregestational diabetes are among the most common metabolic conditions affecting pregnancy, with reported prevalence ranging between 3% and 10% [3].

Maternal hyperglycemia influences fetal development through transplacental transfer of glucose. Elevated maternal glucose levels lead to increased fetal insulin production, which in turn affects fetal growth and metabolic adaptation [4]. This intrauterine environment may result in a range of neonatal outcomes, including metabolic disturbances, altered growth patterns, and respiratory complications.

Evidence from the large-scale study, Hyperglycemia and Adverse Pregnancy Outcomes (HAPO) has demonstrated that even modest elevations in maternal glucose levels are associated with clinically significant changes in neonatal parameters, including increased birth weight, neonatal adiposity, risk of hypoglycemia, and need for intensive neonatal care [5] Infants born to mothers with diabetes are therefore considered a high-risk group requiring careful monitoring during the perinatal period.

Despite extensive literature on neonatal outcomes among infants of diabetic mothers, reported risks and patterns vary across settings, reflecting differences in maternal risk profiles, antenatal care pathways, and glycaemic management strategies [3-5]. Evidence from many regions may not be directly applicable to South India, where population characteristics, comorbidity patterns, and healthcare delivery differ. Moreover, context-specific data describing the distribution and spectrum of neonatal morbidity in this group remain limited. Addressing this gap, the present study was conducted to comprehensively describe neonatal outcomes, including metabolic, respiratory, hematological, and anthropometric parameters, along with perinatal factors and maternal demographic and clinical characteristics, among infants of diabetic mothers in a tertiary care setting in South India.

## Materials and methods

Study design and setting

This was a prospective hospital-based descriptive study conducted in the Department of Pediatrics and Neonatology at a tertiary care hospital in South India over one year (September 2024 to August 2025).

Study population

The study included all live-born infants delivered within the institution to mothers diagnosed with gestational diabetes mellitus or pregestational diabetes. The inclusion and exclusion criteria are presented in Table 1.

**Table 1:** Inclusion and exclusion criteria Outborn infants were excluded to ensure uniformity in antenatal, intrapartum, and immediate neonatal care practices, as variations in referral timing, prior management, and stabilization protocols could significantly influence neonatal outcomes. This approach was intended to reduce heterogeneity and improve internal validity.

Criteria type	Description
Inclusion	Inborn infants of mothers with gestational or pregestational diabetes
Inclusion	Mothers who provided informed consent
Exclusion	Out-born infants
Exclusion	Non-consenting mothers

Sample size

All eligible mother-infant pairs in which the mothers had diabetes and delivered during the study period were included. A total of 161 participants were enrolled. As this was a prospective descriptive study, complete enumeration of all eligible cases during the study period was performed, and no formal sample size calculation was undertaken.

Study tool

Data were collected using a structured, predesigned proforma developed specifically for this study, comprising three sections.

The first section captured maternal information obtained from antenatal records and hospital case files, including maternal age, parity, type of diabetes, and treatment modality. The selected comorbidities (e.g., hypothyroidism, anemia, hypertensive disorders) were elucidated due to their known association with gestational diabetes and their potential independent or additive influence on neonatal outcomes. Maternal height (cm) and weight (kg), recorded during the first trimester visit, were used to calculate body mass index (BMI), which was subsequently classified according to the Asian-specific World Health Organization criteria [6]. Information regarding diabetes in previous pregnancies was not consistently available and was therefore not included in the analysis.

The second section documented perinatal and neonatal characteristics, including mode of delivery, birth weight, gestational age (categorized as small for gestational age (SGA), appropriate for gestational age (AGA), or large for gestational age (LGA)), Apgar scores at one and five minutes, and neonatal anthropometric measurements.

The third section comprised clinical assessment findings and outcome variables, including metabolic, respiratory, hematological, and neurological parameters, along with details of therapeutic interventions administered during the hospital stay. Clinical outcomes were categorized as follows: metabolic - hypoglycemia, hypocalcemia, hypomagnesemia, and metabolic acidosis; respiratory - respiratory distress, transient tachypnoea of the newborn, and requirement of respiratory support; hematological - polycythemia, thrombocytopenia, and anemia.

Operational definitions

BMI was calculated using first-trimester height and weight (kg/m²) and classified based on Asian-specific cut-offs as per the World Health Organization guidelines: underweight (<18.5 kg/m²), normal (18.5-22.9 kg/m²), overweight (23.0-24.9 kg/m²), and obese (≥25.0 kg/m²) [6]. Neonatal anthropometric measurements, including birth weight, crown-heel length, and head circumference, were recorded within one hour of birth using standardized techniques. Classification into small, appropriate, and large for gestational age was performed using INTERGROWTH-21st standards [7]. 

Neonates were monitored from birth until discharge, typically covering the first seven days of life. Blood glucose monitoring was performed at regular intervals as per institutional protocol, particularly within the first 24-72 hours. Early initiation of breastfeeding (within the first hour of life) and timely interventions for metabolic abnormalities were routinely practiced.

At-risk neonates underwent blood glucose screening within one to two hours after birth. Subsequent monitoring was performed every two to four hours during the first 12-24 hours of life, or until at least three consecutive normal pre-feed glucose values were achieved. In our setting, glucose measurements were routinely performed every two hours during the initial 12 hours as part of institutional protocol. Neonatal hypoglycemia was defined as a blood glucose level <45 mg/dL, based on operational thresholds recommended by expert consensus, every two hours till 12 hours after birth [8,9]. 

Hyperbilirubinemia was defined as total serum bilirubin levels reaching or exceeding age-specific phototherapy thresholds according to the National Institute for Health and Care Excellence nomogram (approximately ≥10-12 mg/dL at 24 hours, ≥13-15 mg/dL at 48 hours, and ≥15-18 mg/dL at 72 hours in term neonates) [10]. Polycythemia was defined as a venous hematocrit >65% [11]. Hypomagnesemia was defined as a serum magnesium concentration <1.5 mg/dL (0.62 mmol/L), in accordance with standard neonatal reference values [12]. Respiratory distress was identified based on clinical features including tachypnoea, chest retractions, or grunting, and/or requirement for respiratory support, consistent with World Health Organization recommendations [13].

Statistical analysis

Data were entered in Microsoft Excel and analyzed using SPSS version 23 (IBM Inc., Armonk, New York). As this was a prospective descriptive study, continuous variables were summarised using mean and standard deviation, while categorical variables were expressed as frequencies and percentages.

Ethical considerations

Ethical approval was obtained from the institutional ethics committee (IECHS/IRCHS/No/557). Written informed consent was obtained from all participants.

## Results

**Figure 1:**
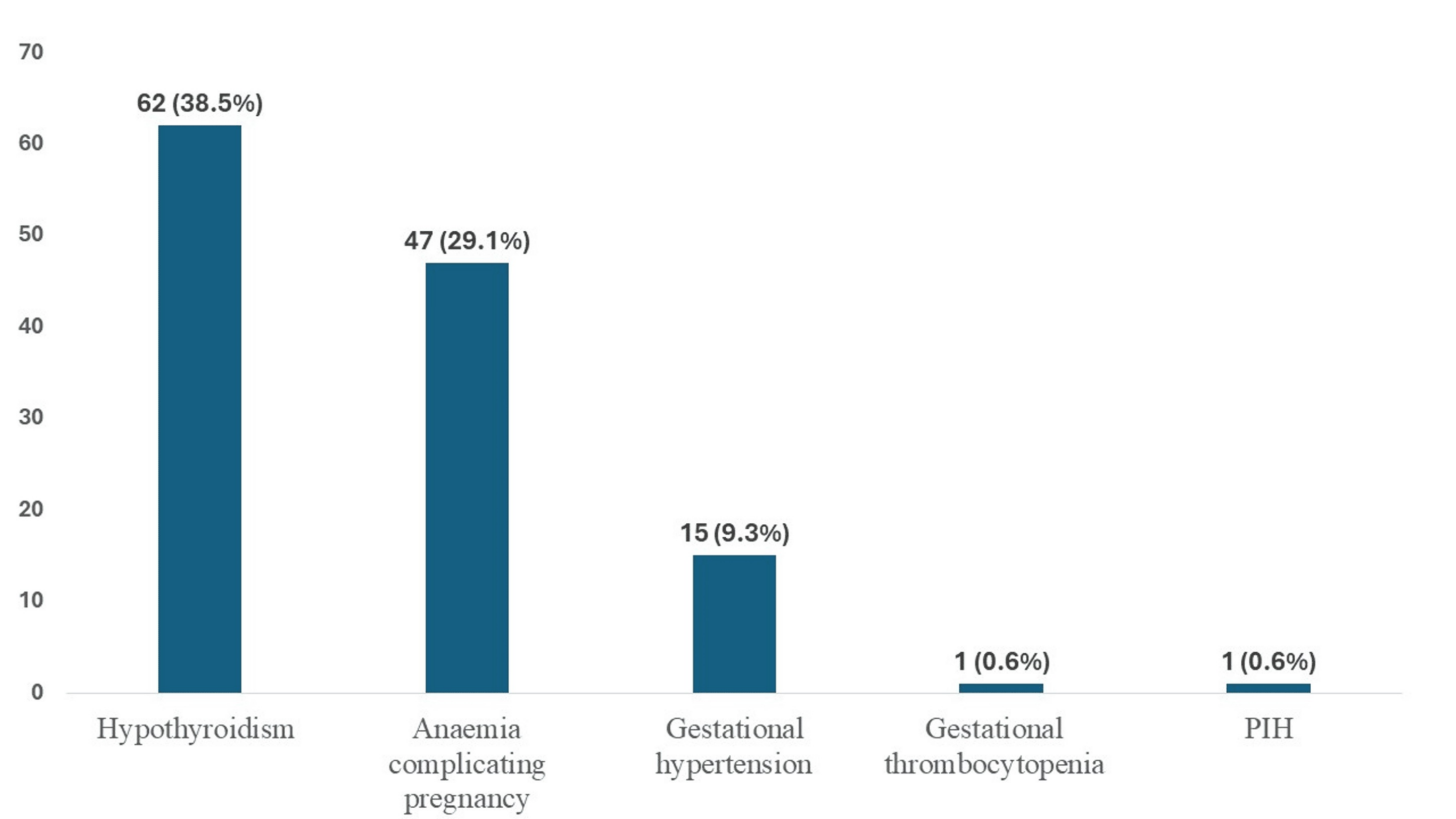
Bar chart showing the comorbidities of GDM mothers (n=161) GDM - gestational diabetes mellitus; PIH - pregnancy-induced hypertension

A total of 161 mother-infant pairs, as per the inclusion criteria, were included, with a mean age of 28.8 ± 4.9 years. Most women were multigravida (91, 56.5%). A considerable proportion were classified as pre-obese or obese (127, 78.9%). Gestational diabetes mellitus constituted 146 (90.7%) cases, while pregestational diabetes accounted for 15 (9.3%). Insulin therapy was used in 77 (47.8%) participants. Comorbidities were present in 108 (67.1%) mothers, with hypothyroidism and anemia being the most common (Table 2, Figure 1).

**Table 2:** Distribution of maternal characteristics among study participants (n=161) DM - diabetes mellitus; GDM - gestational diabetes mellitus; OHA - oral hypoglycaemic agents

Variables	Category	Frequency (n)	Percentage (%)
Maternal age (years)	Mean ± SD	28.8 ± 4.9	—
Religion	Hindu	87	54.0
	Christian	55	34.2
	Muslim	19	11.8
Gravidity	G1	70	43.5
	G2	61	37.9
	≥G3	30	18.6
Parity	Primipara	89	55.3
	Multipara	72	44.7
BMI (kg/m²)	Mean ± SD	29.4 ± 5.4	—
BMI category	Underweight/Normal	34	21.1
	Pre-obese	72	44.7
	Obese	55	34.2
Type of DM	GDM	146	92
	Pregestational DM	15	9
Type of treatment	Diet	48	29.8
	OHA	21	13.0
	Insulin	77	47.8
	Insulin + OHA	15	9.3
Comorbidities	Present	108	67.1
	Absent	53	32.9

Most deliveries occurred at term (143, 88.8%). Cesarean section was the predominant mode of delivery, accounting for 104 (64.6%) cases. Apgar scores were generally satisfactory, with 160 (99.4%) neonates scoring ≥7 at five minutes (Table 3).

**Table 3:** Perinatal characteristics of the study population (N=161) PROM - prelabor rupture of membranes

Parameter	Category	Frequency (n)	Percentage (%)
Gestational age at birth	<32 weeks	1	0.6
	32-33 weeks	3	1.8
	34-36 weeks	14	8.7
	37 weeks	50	31.0
	≥38 weeks	93	57.8
Mode of delivery	Elective cesarean	61	37.9
	Emergency cesarean	43	26.7
	Instrumental delivery	11	6.8
	Normal vaginal delivery	46	28.6
Labour characteristics	Oxytocin augmentation	66	40.9
	PROM >12 hours	6	3.7
	Meconium-stained liquor	17	10.6
APGAR score – one minute	<3	2	1.2
	4–6	8	5.0
	≥7	151	93.8
APGAR score – five minutes	<3	0	0.0
	4–6	1	0.6
	≥7	160	99.4

The mean birth weight was 3.1 ± 0.5 kg. Most infants were appropriate for gestational age (124, 77.0%), while 20 (12.4%) were large and 17 (10.5%) were small for gestational age. Anthropometric parameters such as length, head circumference, and chest circumference were within expected ranges for most neonates (Table 4).

**Table 4:** Neonatal baseline characteristics (N=161) GA - gestational age

Characteristic	Category	Frequency (n)	Percentage (%)
Sex of the baby	Male	85	52.8
	Female	76	47.2
Birth weight (kg)	1.5–1.999	3	1.8
	2.0–2.499	15	9.3
	2.5-2.999	52	32.3
	3.0-3.499	63	39.1
	3.5-3.999	23	14.3
	≥4.0	5	3.1
Mean birth weight (kg)	3.1 ± 0.5	—	—
Birth weight for gestational age (Intergrowth)	Appropriate for GA (AGA)	124	77.0
	Small for GA (SGA)	17	10.5
	Large for GA (LGA)	20	12.4
Length at birth	<45 cm	9	5.5
	46–50 cm	127	78.9
	51-54 cm	25	15.5
Chest circumference	<32 cm	106	65.8
	33-36 cm	54	33.5
	>37 cm	1	0.6
Head circumference	<33 cm	30	18.6
	33-36 cm	126	72.4
	≥37 cm	5	3.1

Hypoglycemia was observed in 22 (13.6%) neonates, making it the most common metabolic abnormality. Hypomagnesemia and metabolic acidosis were each observed in five (3.0%) neonates, while hypocalcemia was noted in four (2.4%) (Table 5).

**Table 5:** Neonatal congenital, metabolic, and hematological findings (N=161) CTEV - congenital talipes equinovarus; CNS - central nervous system

Characteristic	Category	Frequency (n)	Percentage (%)
Congenital anomalies	Renal pelvic ectasis	3	1.8
	CTEV	3	1.8
	Erb's palsy	3	1.8
	Congenital heart defects	2	1.2
	Undescended testis	2	1.2
	Malformed pinna	1	0.6
	CNS malformations	1	0.6
Metabolic complications	Hypoglycemia	22	13.6
	Hypomagnesaemia	5	3.0
	Early metabolic acidosis	5	3.0
	Hypocalcemia	4	2.4
Hematological complications	Thrombocytopenia	6	3.6
	Polycythemia	2	1.2
	Anemia	1	0.6

Hyperbilirubinemia was the most common clinical condition, affecting 35 (21.7%) neonates. Respiratory distress was observed in 25 (15.5%) infants, with transient tachypnoea of the newborn identified as the predominant cause, accounting for 21 (84.0%) of these cases. Neurological complications included seizures in four (2.4%) and hypoxic-ischaemic encephalopathy in two (1.2%) neonates (Table 6).

**Table 6:** Neonatal clinical complications, causes of respiratory distress, and treatment interventions (N=161) CPAP - continuous positive airway pressure; PRBC - packed red blood cells

Characteristic	Category	Frequency (n)	Percentage (%)
Clinical complications	Neonatal hyperbilirubinemia	35	21.7
	Respiratory distress syndrome	25	15.5
	Neonatal seizures	4	2.4
	Hypoxic-ischaemic encephalopathy	2	1.2
	Shock	1	0.6
	Delayed passage of meconium	4	2.4
	Cephalhematoma	5	3.0
Causes of respiratory distress (n=25)	Transient tachypnea of the newborn	21	84
	Meconium aspiration syndrome	1	4
	Hyaline membrane disease	1	4
	Pneumonitis	1	4
Treatment interventions for respiratory distress (n=25)	Antibiotics	22	88
	IV fluids	19	76
	Mechanical ventilation (IPPV)	4	16
	CPAP	3	12
	Anti-epileptics	3	12
	Hydrocortisone	3	12
	Aminophylline	2	8
	Inotropes	1	4
	PRBC transfusion	1	4
	Platelet transfusion	1	4

## Discussion

The present study provides a comprehensive description of maternal and neonatal outcomes among infants born to mothers with diabetes, highlighting the continued burden of neonatal morbidity in this high-risk population. Diabetes in pregnancy is a well-recognized metabolic disorder with implications extending beyond maternal health, significantly influencing fetal growth, metabolic adaptation, and early neonatal outcomes [1,2,14]. The pathophysiological basis of these complications lies in maternal hyperglycemia leading to fetal hyperinsulinemia, which in turn affects multiple organ systems [3,4].

In this study, the mean maternal age was 29.1 ± 4.8 years, with a predominance of gestational diabetes mellitus (91%), consistent with global and Indian trends indicating increasing GDM prevalence [3,15]. The high proportion of overweight and obese mothers (approximately 78.9%) aligns with existing evidence that maternal adiposity contributes to insulin resistance and the development of gestational diabetes [4]. Similar maternal profiles have been reported by Reddy et al. and Thomas et al., where the majority of affected women were in their late twenties and had comparable BMI distributions [15,16]. However, the higher prevalence of comorbidities such as hypothyroidism (38.5%) and anemia (29.1%) observed in the present study contrasts with some earlier reports [17,18], possibly reflecting regional variations in nutritional status and antenatal screening practices.

The perinatal profile in this cohort showed that the majority of deliveries occurred at term (88.8%), which is consistent with findings from Opara et al. and Jadhav et al., where term deliveries predominated despite maternal diabetes [19,20]. The high rate of cesarean section (64.6%) observed in this study is comparable to previous reports [17,18]. Whereas this rate is higher than the reported overall cesarean section rate in India (approximately 21-25% based on national data), reflecting the higher obstetric risk associated with diabetic pregnancies, including concerns of fetal macrosomia, intrapartum complications, and the need for closer monitoring. 

Neonatal anthropometric findings demonstrated that most infants were appropriate for gestational age (77.0%), with smaller proportions of large-for-gestational-age (12.4%) and small-for-gestational-age (10.5%) infants. This distribution is consistent with studies by Gopal et al. and Nili et al., where AGA infants predominated despite maternal diabetes [21,22]. Although diabetes is typically associated with large-for-gestational-age infants, SGA may occur due to factors such as placental insufficiency, coexisting maternal conditions (e.g., hypertension, anemia), or stringent glycaemic control limiting fetal growth. However, earlier studies have reported higher rates of macrosomia [16,23], suggesting that improved antenatal care and glycaemic management in the present setting may have contributed to a relatively lower proportion of LGA infants.

Metabolic complications were a prominent feature in this cohort, with hypoglycemia observed in 13.6% of neonates. This finding is in agreement with previous studies identifying hypoglycemia as the most common metabolic disturbance among infants of diabetic mothers [16,23,24]. However, the incidence in the present study appears lower than that reported in some earlier cohorts [12,17], where higher rates were attributed to poor glycaemic control and delayed initiation of feeding. The observed decline in hypoglycemia from 13.7% at one hour to 1.9% at three hours in this study reflects early metabolic stabilization, which is consistent with physiological adaptation patterns described in standard neonatal literature [14]. The relatively lower incidence observed in this study may be attributed to institutional practices such as early initiation of breastfeeding, structured glucose monitoring protocols, prompt correction of hypoglycemia, and improved antenatal glycaemic control including better antenatal glycaemic control, which reduces fetal hyperinsulinemia and its inhibitory effect on surfactant synthesis; timely obstetric decision-making, minimizing prolonged labour and fetal distress; and increased use of planned deliveries in controlled settings.

Other metabolic abnormalities, such as hypocalcemia (2.4%) and hypomagnesemia (3.0%), were relatively infrequent compared to earlier studies [19,24], where higher incidences were reported. This difference may be explained by improved neonatal monitoring and early initiation of feeding practices in the current setting.

Hematological complications, particularly hyperbilirubinemia (21.7%), were among the most common clinical findings. This is comparable to findings from Hussain et al. and Anjum et al., who also reported hyperbilirubinemia as a frequent neonatal condition in this population [23,24]. However, the incidence remains within the expected physiological range for transitional neonatal adaptation, suggesting that while common, it may not always represent severe pathology.

Respiratory morbidity was observed in 15.5% of neonates, with transient tachypnoea of the newborn accounting for the majority (12.6%). This is consistent with previous reports indicating delayed lung maturation in infants of diabetic mothers due to the inhibitory effects of hyperinsulinemia on surfactant production [22,24]. Compared to earlier studies [22-24], the present study demonstrated lower rates of severe respiratory conditions such as meconium aspiration syndrome and hyaline membrane disease, which may reflect improved obstetric and neonatal care practices.

Congenital anomalies were relatively uncommon in this cohort, with individual anomalies occurring in less than 2% of cases. This contrasts with some earlier studies reporting higher rates of congenital malformations, particularly in pregestational diabetes [23,25]. The lower frequency observed here may be attributed to better preconception care and early antenatal screening. Overall, the lower incidence of severe complications observed in this cohort suggests that early diagnosis and structured management of maternal diabetes can contribute to improved neonatal outcomes.

Overall, the present study indicates that although most infants of diabetic mothers achieved term delivery and appropriate growth parameters, neonatal morbidity remains non-trivial and clinically relevant. The comparatively lower frequency of severe outcomes such as macrosomia, hypoglycemia, and respiratory distress, relative to earlier reports, suggests incremental improvements in antenatal detection and glycaemic management. However, the continued occurrence of metabolic disturbances and hyperbilirubinemia indicates that optimal glycaemic control does not fully mitigate neonatal risk. These findings highlight a persistent gap between improved maternal management and complete neonatal risk reduction. From a clinical perspective, this underscores the need for sustained emphasis on early identification, stringent glycaemic monitoring, and protocol-driven neonatal surveillance. From a public health standpoint, the results suggest that while current strategies are effective in reducing severity, they may be insufficient to eliminate morbidity, warranting further refinement of integrated care pathways and closer evaluation of residual risk factors in this population.

This study has several strengths, including its prospective design and comprehensive neonatal assessment using standardized definitions, which enhance internal validity. However, certain limitations should be acknowledged. The single-center setting may limit generalisability, and the descriptive design precludes causal inference. Additionally, although the sample size was adequate for descriptive analysis, it restricted detailed subgroup evaluation, limiting the ability to draw causal inferences. Long-term neonatal outcomes were not assessed, which remains an important area for future research. The exclusion of outborn infants may introduce selection bias and limit generalizability. Furthermore, the lack of subgroup analysis (e.g., gestational vs pregestational diabetes or treatment modalities) restricts deeper interpretation. Data regarding diabetes in previous pregnancies were not consistently available and could not be analyzed.

## Conclusions

This study provides a detailed description of neonatal outcomes among infants of diabetic mothers. While the majority of GDM mothers achieved favorable birth outcomes, neonatal morbidity remains clinically significant. Hypoglycemia and hyperbilirubinemia emerged as the most frequent conditions requiring early monitoring, with additional contributions from respiratory morbidity and less frequent metabolic disturbances such as hypocalcemia and hypomagnesemia. Although the overall incidence of severe complications appears lower than in earlier reports, these findings indicate that neonatal risk persists despite advances in antenatal care and glycaemic management.

These results underscore the importance of systematic neonatal surveillance, early metabolic screening, and prompt initiation of supportive care in infants born to mothers with diabetes. Strengthening integrated maternal-neonatal care pathways, including optimized glycaemic control during pregnancy and standardized postnatal monitoring protocols, remains essential to minimize preventable morbidity.
